# Impact of gene expression data pre-processing on expression quantitative trait locus mapping

**DOI:** 10.1186/1753-6561-1-s1-s153

**Published:** 2007-12-18

**Authors:** Aurelie Labbe, Marie-Paule Roth, Pierre-Hugues Carmichael, Maria Martinez

**Affiliations:** 1 Département de Mathématiques et de Statistique, Université Laval, Québec, G1K 7P4, Canada; 2 Centre de Recherche Université Laval Robert Giffard, Québec, G1K 7P4, Canada; 3 INSERM U563, Centre de Physiopathologic de Toulouse Purpan Toulouse, F-31300, France; Université Toulouse, III Paul-Sabatiere, Toulouse, F-31400, France

## Abstract

We evaluate the impact of three pre-processing methods for Affymetrix microarray data on expression quantitative trait locus (eQTL) mapping, using 14 CEPH Utah families (GAW Problem 1 data). Different sets of expression traits were chosen according to different selection criteria: expression level, variance, and heritability. For each gene, three expression phenotypes were obtained by different pre-processing methods. Each quantitative phenotype was then submitted to a whole-genome scan, using multipoint variance component LODs. Pre-processing methods were compared with respect to their linkage outcomes (number of linkage signals with LODs greater than 3, consistencies in the location of the trait-specific linkage signals, and type of *cis*/*trans*-regulating loci). Overall, we found little agreement between linkage results from the different pre-processing methods: most of the linkage signals were specific to one pre-processing method. However, agreement rates varied according to the criteria used to select the traits. For instance, these rates were higher in the set of the most heritable traits. On the other hand, the pre-processing method had little impact on the relative proportion of detected *cis *and *trans*-regulating loci. Interestingly, although the number of detected *cis*-regulating loci was relatively small, pre-processing methods agreed much better in this set of linkage signals than in the *trans*-regulating loci. Several potential factors explaining the discordance observed between the methods are discussed.

## Background

Evidence for heritability of mRNA levels has been observed in several organisms like the mouse, yeast, and human [1-3]. Therefore, it is possible to consider transcript levels measured using DNA microarrays as quantitative traits and localize the genes controlling them by quantitative trait locus (QTL) analyses. However, one of the challenges is to remove optical noise and take into account nonspecific hybridization in the microarray experiments. In addition, in the Affymetrix system, each gene is represented by 11–20 Perfect Match (PM) and Mismatch (MM) pairs of probes, each probing a different region of the mRNA transcript, typically within 600 base pairs of the 3' end [4]. It is also important to find a way to combine the 11–20 probe pair intensities for a given gene into a single measure. Several data pre-processing methods are now available. Of several such proposed methods, three have commonly been employed: the Robust Multiarray Average (RMA), GeneChip RMA (GCRMA), and the Microarray Analysis Suite 5 (MAS5) methods. A detailed description of these methods can be found elsewhere [5-7]. These three methods convert probe-level data to expression values through the following sequence: 1) background correction, 2) normalization, and 3) summarizing the probe set values into one expression measure. Regarding the background correction step, MAS5 uses the MM probes to adjust the PM probes for probe-specific nonspecific binding [5]. On the other hand, RMA ignores the MM intensities and performs a global background correction [6]. GCRMA employs a hybrid approach and uses the probe sequence information released by Affymetrix to compute an affinity measure and describe background noise. For Step 2, RMA and GCRMA use the same normalization method based upon intensity quantiles, which imposes the same empirical distribution of intensity to each array. In MAS5, a baseline array is chosen and all the other arrays are scaled to have the same mean intensity as this array. Finally, RMA and GCRMA use the same probe summary method, based on a robust linear model. In MAS5, the probe summary is based on a robust average method.

Different DNA chip pre-processing methods have been shown to influence measures of gene expression [6,8,9]. Therefore, the choice of a pre-processing method significantly affects linkage results obtained in genomic analyses [10]. However, whether or not only concordant results should be further investigated is still debated (see Petretto et al. [11] and Chesler et al. [12] in their reply to Williams et al. [10]). In this paper, we focus on comparing the degree of concordance between pre-processing methods, according to the characteristics of the traits selected for linkage analysis.

## Methods

The study is based on all 14 three-generation CEPH (Centre d'Etude du Polymorphisme Humain) Utah families. Gene expression levels in lymphoblastoid cells of 194 individuals have been obtained using the Affymetrix Human Focus Arrays that contain probes for 8792 transcripts. Details regarding the microarray experiments are given in Morley et al. [3].

### Data pre-processing

Transcript expression data were obtained using the three methods described above: MAS5, RMA, and GCRMA. Expression levels in 82 individuals with technical replicates were averaged over replicates. All subsequent analyses were performed on log_2_-transformed values.

### Choice of the traits for linkage analysis

It may be appropriate to restrict linkage analysis to the traits that are expressed in the target tissue and show detectable variation between individuals. Clearly, this is rarely the case for all the transcripts analyzed on a microarray. For example, Morley et al. [3] chose to analyze less than 50% of the measured traits, according to the ratio of the variances of trait expressions between and within individuals. Here, we first used the detection (Present/Absent) call generated by the Affymetrix MAS5 software to identify transcripts that were not reliably detected. 3727 transcripts were found to be significantly expressed in at least 80% of the arrays (at the significant level of 4%) and are referred in this paper as the "expressed set". The other set of 5065 transcripts is referred as the "non-expressed set". Three groups of genes were selected in the expressed set of genes, based on the distribution of their expression levels measured by the MAS5 preprocessing method in the 194 available individuals: 1) the 100 genes with the highest variance in the measured expression phenotype, 2) the 100 genes with the most heritable expression, and 3) 350 genes chosen at random among the top 50% genes with the largest variance. Note that 8 genes were common to groups 1 and 2, 22 to groups 1 and 3, and 2 to the three groups. In addition, a fourth group consisted of the 100 top most variable genes of the non-expressed set. A total of 3 × 650 expression phenotypes were therefore submitted to linkage analysis.

### Linkage analysis

The loci controlling expression levels (the quantitative traits) of these 650 genes were localized using all autosomal marker data provided in the Problem 1 GAW15 dataset. Multipoint LOD scores were computed using the variance-component linkage test with the software Merlin [13]. In this paper, we define linkage peaks as the highest multipoint LOD within a 20 Mb-interval. In this study, we deemed as "linkage signals" linkage outcomes that have a LOD score ≥ 3, not accounting for the multiple testing problem.

### Criteria to compare pre-processing methods

Several measures were defined in order to compare the three pre-processing methods: 1) the number of linkage signals, 2) the number of traits with at least one linkage signal as well as the number of linkage signals per trait, and 3) the location of the loci controlling gene expression with respect to the position of the gene itself (*cis*- or *trans*-acting loci). A gene was assumed to be *cis*-regulated when the locus controlling its expression level mapped within 10 Mb of the gene itself, and *trans*-regulated otherwise. Finally, results obtained using two different pre-processing methods were considered consistent when the distance between their linkage peaks was less than 20 Mb. Concordance rates between two or more methods were defined as the ratio of the number of consistent linkage peaks between methods over the total number of linkage peaks detected by any of the three methods.

## Results

### Comparison of the linkage signals detected for the three methods

**Figure 1:**
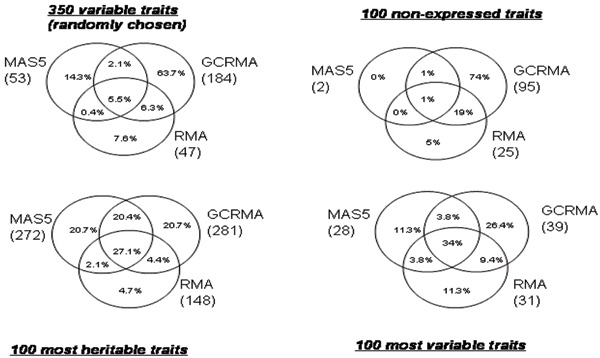
Concordance and discordance rates of the linkage signals between pre-processing methods (with number of linkage signals per method).

Figure 1 gives the number of linkage signals for each pre-processing method in parentheses as well as the concordance rate between methods evaluated as described above. Not surprisingly, more linkage signals were found for the most heritable traits than for those with the highest variance. More linkage signals were detected with GCRMA than with the two other methods, especially in two sets, the set of 350 traits randomly chosen and that of 100 non-expressed traits. In general, less than half of the linkage signals are concordant between two or three methods. However, these rates vary according to the group of genes analyzed: the most important discrepancies were observed for the set of non-expressed genes and the set of the 350 genes chosen at random: 74% and 63.7% of the signals, respectively, were specific to the GCRMA pre-processing method. Furthermore, for the group of 100 non-expressed genes, 2 signals with LOD ≥ 3 were detected with MAS5, whereas 95 and 25 signals were detected with GCRMA and RMA, respectively. For the three other groups, RMA was the pre-processing method that generated the lowest rate of specific signals (from 4.7% to 11.3%).

### Comparison of the number of traits with at least one linkage signal as well as the number of linkage signals per trait

Table 1 shows the number of detected eQTLs and the average number of linkage signals per trait. Again, on average, we observe that loci regulating gene expression were found for more genes when data were pre-processed using GCRMA, especially in two sets of traits. In the set of the 350 traits chosen at random, a high proportion of the linkage signals (64%) were accounted by only two of the expression traits, SYMPK and ARG2. The same trend was observed in the set of non-expressed traits, where 60% of the signals were accounted by the two traits TIE1 and FUT7.

**Table 1:** Number of detected traits (having at least one linkage signal) and mean number of linkage signals per trait

	**No. Traits detected (%)**			**Mean no. of linkage signals by detected trait (range)**		
		
**Set of traits**	**MAS5**	**GCRMA**	**RMA**	**MAS5**	**GCRMA**	**RMA**
**350 random**	37/350 (11%)	45/350 (13%)	36/350 (10%)	1.43 (1–4)	4.08 (1–61)	1.30 (1–3)
**100 most variable**	18/100 (18%)	21/100 (21%)	17/100 (17%)	1.55 (1–4)	1.85 (1–5)	1.82 (1–7)
**100 most heritable**	39/100 (39%)	36/100 (36%)	34/100 (34%)	6.97 (1–90)	7.8 (1–111)	4.35 (1–54)
**100 non-expressed**	2/100 (2%)	14/100 (14%)	7/100 (7%)	1 (1-1)	6.5 (1–30)	3.6 (1–9)

### Comparison of the *cis*-acting and *trans*-acting detected eQTLs

The number of *cis*-, *cis*-*trans*, and *trans*-acting loci detected by each method is given in Table 2. Similar distributions were obtained with all three pre-processing methods. The majority of the detected traits are *trans*-regulated: from 65–68% in the set of non-expressed genes, to 76–89% in the set of 350 variable traits. Nevertheless, for *cis*-regulating signals (results not shown), concordance rates between the three methods were 44% (4/9) for the group of 350 traits chosen at random, 100% (1/1) in the group of non-expressed genes, 73% (11/15) in the group of the most heritable genes, and 60% (3/5) in the group of the most variable traits. Further, concordance rates between GCRMA and RMA were especially high and always greater than 75%. On the other hand, in the remaining set of signals (trans-regulators), much lower concordance rates were obtained (<34%, for all sets of genes studied).

**Table 2:** Number of *cis*-, *cis*/*trans*, and *trans*-regulated traits for each method (proportion among traits having at least one linkage signal)

	*cis*	*cis*/*trans*	*trans*
**350 variable traits**			
**MAS5**	7 (19%)	2 (5%)	28 (76%)
**GCRMA**	4 (9%)	1 (2%)	40 (89%)
**RMA**	3 (8%)	1 (3%)	32 (89%)

**100 most variable traits**			
**MAS5**	1 (50%)	0 (0%)	1 (50%)
**GCRMA**	1 (7%)	0 (0%)	13 (93%)
**RMA**	1 (14%)	0 (0%)	6 (86%)

**100 most heritable traits**			
**MAS5**	2 (11%)	3 (17%)	13 (72%)
**GCRMA**	2 (9.5%)	2 (9.5%)	17 (81%)
**RMA**	3 (18%)	1 (6%)	13 (76%)

**100 non-expressed traits**			
**MAS5**	8 (20%)	6 (15%)	25 (65%)
**GCRMA**	7 (19%)	5 (14%)	24 (67%)
**RMA**	9 (26%)	2 (6%)	23 (68%)

## Discussion

As previously suggested by other studies [6,8,10], our results confirm that pre-processing methods may also affect linkage outcomes. However, this impact depends on the way traits were selected for genetic analysis. Choosing the traits on the basis of a high heritability value led to a minimum discrepancy between methods. Conversely, discrepancies were more important in the group of non-expressed traits or in the group of variable traits chosen at random (note that in this last group, heritability ranges from 0% to 50%). Furthermore, we noticed that the three pre-processing methods agree much better for *cis*-acting than for trans-acting regulators.

Several factors may explain partly why the three methods produce different results. First, as already stated in the Background, the underlying models converting probe level data to expression values are different from one method to another. Although the normalization step is not the same for the three methods, previous work has shown that these differences have little effect relative to that of the background correction, which entails a variance/bias trade-off. Especially, it has been shown that background correction decreases the bias but that naïve background correction procedures, such as MAS5 and RMA, increase the variance [8]. GCRMA is supposed to provide a good balance between accuracy and precision by doing adequate non-specific binding correction. Our study suggests that the large impact of the background correction also applies to eQTL mapping results. Indeed, RMA and GCRMA differ only by the background correction step, yet their concordance rates were not particularly high. Interestingly, Irizarry et al. [8] observed that differences in precision between RMA and GCRMA were higher in the case of genes with low expression, with GCRMA giving the smallest bias. We also observed a greater discordance rate between RMA and GCRMA for the set of non-expressed genes.

Another potential factor for explaining differences between methods is departure from normality of the phenotypic distribution, especially when using a variance-component approach. We found that, in general, GCRMA led to the highest rate of traits failing the Shapiro normality test. Among expressed genes, these rates were 5.4, 0.8, and 1.7% for GCRMA, RMA, and MAS5, respectively (using a Bonferroni correction for multiple testing at level 5%). These rates might explain the large number of linkage signals observed for a few traits with GCRMA. However, it seems unlikely that conflicting eQTL mapping results are mainly due to differences in the gene expression distributions per se.

In conclusion, the true genetic determinants of the studied traits in the GAW Problem 1 data are unknown, preventing us from drawing definite conclusions on the best and more robust pre-processing method. Further, in the context of a genome-scan, high agreement rates across experiments are not expected because most of the linkage signals are likely to be false positives. It is unclear whether it would be sound to use several pre-processing methods in a systematic manner. Such guidelines were proposed recently but remain controversial [10-12]. In our study, we found very poor agreement between pre-processing methods in the set of the non-expressed but most variable genes (i.e., our Set 4), suggesting that filtering genes on their detectable presence in the tissue of interest is also an important step. To filter genes not only on their variability but also on their presence in the tissue analyzed is one of our main messages.

## Competing interests

The author(s) declare that they have no competing interests.
